# AltGosling: automatic generation of text descriptions for accessible genomics data visualization

**DOI:** 10.1093/bioinformatics/btae670

**Published:** 2024-11-14

**Authors:** Thomas C Smits, Sehi L’Yi, Andrew P Mar, Nils Gehlenborg

**Affiliations:** Department of Biomedical Informatics, Harvard Medical School, Boston, MA 02115, United States; Department of Biomedical Informatics, Harvard Medical School, Boston, MA 02115, United States; Department of Biomedical Informatics, Harvard Medical School, Boston, MA 02115, United States; Department of English, University of California, Berkeley, CA 94720, United States; Department of Biomedical Informatics, Harvard Medical School, Boston, MA 02115, United States

## Abstract

**Motivation:**

Biomedical visualizations are key to accessing biomedical knowledge and detecting new patterns in large datasets. Interactive visualizations are essential for biomedical data scientists and are omnipresent in data analysis software and data portals. Without appropriate descriptions, these visualizations are not accessible to all people with blindness and low vision, who often rely on screen reader accessibility technologies to access visual information on digital devices. Screen readers require descriptions to convey image content. However, many images lack informative descriptions due to unawareness and difficulty writing such descriptions. Describing complex and interactive visualizations, like genomics data visualizations, is even more challenging. Automatic generation of descriptions could be beneficial, yet current alt text generating models are limited to basic visualizations and cannot be used for genomics.

**Results:**

We present AltGosling, an automated description generation tool focused on interactive data visualizations of genome-mapped data, created with the grammar-based genomics toolkit Gosling. The logic-based algorithm of AltGosling creates various descriptions including a tree-structured navigable panel. We co-designed AltGosling with a blind screen reader user (co-author). We show that AltGosling outperforms state-of-the-art large language models and common image-based neural networks for alt text generation of genomics data visualizations. As a first of its kind in genomic research, we lay the groundwork to increase accessibility in the field.

**Availability and implementation:**

The source code, examples, and interactive demo are accessible under the MIT License at https://github.com/gosling-lang/altgosling. The package is available at https://www.npmjs.com/package/altgosling.

## 1 Introduction

People with blindness and low vision (BLV) often face barriers when interacting with web content (Kim *et al.* 2021). BLV people commonly rely on screen readers (e.g. JAWS, NVDA, and Apple’s Voice Over) (https://webaim.org/projects/screenreadersurvey7/), assistive technologies that describe web elements through speech. The World Wide Web Consortium (W3C) has adopted the Web Content Accessibility Guidelines (WCAG) for web accessibility. For images, content creators should provide alt text (alternative text) for screen reader users. For complex images, the WCAG 2.1 requires both the alt text and a longer textual description (https://www.w3.org/WAI/tutorials/images/complex/). In addition, SVG-based images can include ARIA (Accessible Rich Internet Applications) elements to provide extra context. However, many images, including scientific figures, lack informative descriptions (Sharif *et al.* 2021, L’Yi *et al.* 2023), partially due to a lack of awareness and support. For complex images, manually writing accurate and complex descriptions is not a trivial task. Furthermore, descriptions for auto-generated and dynamically created images, such as interactive visualizations, cannot be manually created. Therefore, the automatic creation of text descriptions is beneficial for web accessibility.

This automation can be achieved with computer vision strategies, focusing on detecting elements on pixel-based graphics (e.g. figures in PNG), and with structured visualizations, such as SVG-based images or grammar-based visualizations (Lundgard and Satyanarayan 2022). Both domains have seen several contributions, including EvoGraphs (Sharif and Forouraghi 2018) and VoxLens (Sharif *et al.* 2022). While promising, current efforts focus on a small set of chart types (e.g. bar charts and pie charts) or limited data dimensionality (Kim *et al.* 2021), not supporting complex images such as those in the field of genomics. BrailleR detects features in R graphs (Godfrey *et al.* 2023), though due to its concatenated flat-text output, the descriptions for more complicated graphs are long and hard to navigate (Jung *et al.* 2022). Notably, Olli (https://mitvis.github.io/olli/) is a visualization library that creates a keyboard navigable tree view with adapters for Vega, Vega-Lite, and Observable Plot. However, these libraries are not suited for genomics data visualization due to a lack of support for genomics data operations and file formats, support for diverse layouts, or multi-view linking (L’Yi *et al.* 2022). Artificial Intelligence (AI) has also sparked a range of new tools, many of which are image-based, such as Amazon Rek and Google Cloud Vision AI (Leotta *et al.* 2022). With the introduction of Large Language Models (LLMs), AI can also be used in the domain of structured visualizations. However, there are currently no tools in the domain of biomedical data, and in particular genomics, that fill the unmet need for accessibility.

Only 3.4% of the genomics workforce reported having disabilities (reported by the American Society of Human Genetics, https://www.ashg.org/wp-content/uploads/2022/11/WorkforceSurveyReport_Report_FINAL2.pdf), which notably differs from the US adult population (27%) (Varadaraj *et al.* 2021). The vast majority (90%) of biomedical resources fail accessibility standards (L’Yi *et al.* 2023). Considering that biomedical visualizations are key to accessing biomedical knowledge and detecting new patterns in large datasets (Nusrat *et al.* 2019), increasing accessibility of visualization is essential for more inclusive biomedical research and education. To encompass a wide variety of genomics visualizations created as structural visualizations, we use Gosling, a grammar-based data visualization toolkit for genome-mapped data (L’Yi *et al.* 2022). We will use the term “genomics data” to refer to genome-mapped data for simplicity. Characterized by its notable expressiveness, the Gosling grammar can capture and express almost all existing genomics and novel visualizations, focusing on interaction and data scalability.

Here, we present AltGosling, an automatic text description generator for accessible genomics data visualizations based on Gosling. Genomic visualizations tend to be complex and visualize large datasets (Nusrat *et al.* 2019, L’Yi *et al.* 2022). This brings additional challenges for creating effective text descriptions, such as large descriptions to capture the amount of data and large compositions, and updating descriptions for interactivity. With AltGosling, we tackle a number of these challenges by making the descriptions modular, concise and accurate. By relying on Gosling’s internal rendering, we extract additional information that is not directly available in the visualization specification. AltGosling separates its feature extraction and rendering of text descriptions, providing flexibility and scalability to users. Using the four-level semantic model proposed by Lundgard and Satyanarayan (Lundgard and Satyanarayan 2022), we focus visual encoding (Level 1) and statistical information (Level 2) descriptions in a hierarchical view, where users can determine the specificity level they consume, easing the access to large complex descriptions. We iterate over our design in a co-design process with a screen reader user. To assess the usefulness of AltGosling, we demonstrate AltGosling’s text descriptions for various genomics data visualizations, followed by a comparison to state-of-the-art tools and models in generating alt text of genomics data visualizations.

## 2 Materials and methods

In this section, we first describe design considerations that guided us in designing and developing AltGosling. We then describe our iterative co-design process with a screen reader user, followed by the detailed approach in AltGosling to generate various types of textual descriptions.

We highlight that leading guidelines from W3C on creating text descriptions are focused on static visualizations. Generating descriptions for interactive visualizations is an underexplored area of study. Interactive visualizations in the biomedical domain have two key functions: (i) conveying information about patterns in the data and (ii) allowing exploration and discovery of new patterns (Nusrat *et al.* 2019). Adhering to leading guidelines covers this first function. In order to support the second function, descriptions should aid in the task of exploring new patterns. In addition to conveying information about biomedical data, similar to static visualizations, interactive visualizations are key for discovering new patterns. An effective text description should assist both goals. Therefore, aside from conveying information based on leading guidelines, descriptions should aid in the task of exploring and discovering new patterns.

### 2.1 Design principles

Focusing on accessibility for users with blindness or low vision (BLV users), we identified seven design principles that are important for providing textual descriptions of genomics data visualizations after reviewing existing frameworks and studies.

1. **Completeness**: The descriptions should be as complete as possible for understanding genomics data visualizations. We focus on the two most important types of content identified from a four-level text description model (Lundgard and Satyanarayan 2022): visual encoding (Level 1) and statistical information (Level 2).
2. **Accuracy**: We aim for our descriptions to be accurate and not contain wrong information (e.g. conveying a wrong axis or chart type).
3. **Conciseness**: The descriptions should be concise, and reading them should not bear a significant cognitive load (Zong *et al.* 2022).
4. **Structure**: The text’s structure greatly affects the cognitive load (Zong *et al.* 2022). To make descriptions follow a logical flow (Jung *et al.* 2022), we use the top-down approach, starting with a chart type and other important features, and following with more detailed properties.
5. **Navigability**: Because genomics data visualizations are usually complex and contain large-scale information, descriptions to explain them could become long. Long plain text can be hard to navigate (Zong *et al.* 2022), lowering efficiency in getting the desired information from the descriptions. Therefore, descriptions should be easily navigable to help gain information more efficiently.
6. **Modularity**: We aim for our tool to be modular to accommodate an iterative design process that meets various use cases.
7. **Inclusivity**: We aim not to design for people with disabilities but with them. This is highly important, as refraining from doing so can help perpetuate stereotypes and further marginalize people with disabilities (Lundgard *et al.* 2019).

### 2.2 Co-design process

We designed AltGosling in an iterative co-design process with a screen reader user. After an exploratory phase of four months of retrieving automatic descriptions, we iterated over the design principles. Then, an initial version of AltGosling was created focusing on flexibility. In this phase, the authors regularly met together for 10 months to discuss and implement the design choices in AltGosling based on the aforementioned design principles. We then iteratively improved AltGosling for two months by collecting reviews from one of the co-authors, using a set of genomics data visualization examples. This co-author is an undergraduate student who has been totally blind since age 19. They have no exposure to genomics through their undergraduate program.

The co-author used the JAWS screen reader in Google Chrome. In subsequent weeks, they reviewed a singular example at a time, after which these evaluations were discussed in meetings via Zoom with the other co-authors (who have full vision). In these discussions, we planned and outlined the changes for the second iteration, which we present in this manuscript. We include the conclusions from these discussions in Section 3.2.

### 2.3 AltGosling approach

**Figure 1.**
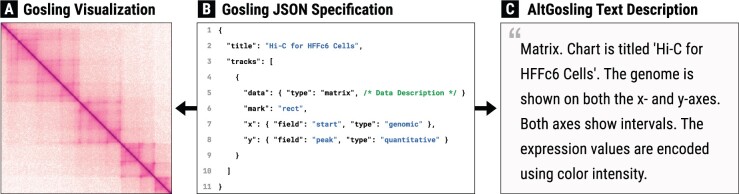
Relationship between AltGosling and Gosling. The logic-based algorithm of AltGosling extracts important features of Gosling visualization (L’Yi *et al.* 2022) (A) from its corresponding JSON specification (B) to automatically generate natural language description (C). Screen readers can use the resulting description (C) to explain visualization to blind and low vision (BLV) users.

We focused on the Gosling grammar-based toolkit to automatically create text descriptions of genomics data visualization using the Gosling specifications (L’Yi *et al.* 2022). Gosling can be used in a number of different ways: through the Gosling.js JavaScript library, the Gos Python package (Manz *et al.* 2023), and a preliminary R package (https://github.com/gosling-lang/grosling), as well as in the online editor (https://gosling.js.org). In each instance, the visualization (Fig. 1A) is based on a JSON specification defining how referenced data should be displayed (Fig. 1B). Gosling depends on HiGlass for its data retrieval (Kerpedjiev *et al.* 2018). For static visualizations, it retrieves the genomics data in HiGlass tiles once. For interactive visualizations, it retrieves the genomics data upon each update on the visualization (e.g. through zoom and pan) and sends the data over to the Gosling JavaScript API.

**Figure 2.**
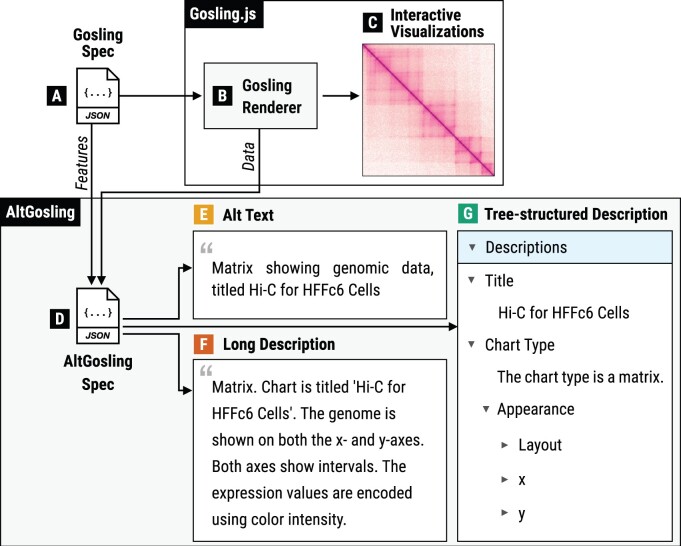
A schematic representation of the AltGosling approach. AltGosling (D) extracts information about visual representations and underlying data of a genomics data visualization (C) from the Gosling JSON specification (A) and the Gosling renderer (B). Based on the extracted features, AltGosling offers alternative text (“alt text”), long descriptions, and tree-structured keyboard-navigable descriptions. The separation of feature extraction (D) and the rendering of textual descriptions (E–G) offers flexibility in delivery.

AltGosling depends on the Gosling specification and the Gosling JavaScript API (Fig. 2). Using information from these components, AltGosling creates its own specification that captures all important features and corresponding text descriptions (Fig. 2D). Compared to the Gosling specification, AltGosling specifications are less nested and contain the entire information for generating text descriptions. The AltGosling specification is used to deliver the plain text descriptions (Figs 1C and 2E–F) as well as the navigable tree-structured descriptions (Fig. 2G). The rendering logic of AltGosling defines what properties from the AltGosling specification should be shown. This separation of information retrieval (Fig. 2D) and rendering logic (Fig. 2E–G) allows for easy variation in delivery methods. For example, developers can directly use the AltGosling specification to create custom text descriptions that meet their use cases.

### 2.4 Text descriptions

#### 2.4.1 Processing gosling specifications

Much of Gosling’s flexibility is provided by its nested structure. Briefly, specifications are organized into ***tracks*** (defining a dataset and a visual encoding of the data) and ***views*** (defining a genomic range and how one or multiple tracks are arranged relative to each other), where a view is a combination of tracks that are grouped in a particular way. Properties can be specified for a singular track, or for all tracks in a view, by varying the level of the property. By overlaying tracks representing the same or different datasets on top of each other, more complex visualization can be created from basic graphic primitives such as lines, boxes, and circles.

Gosling processes its input JSON specification to fill in inherited values and defaults for all its tracks. AltGosling relies on this processing, retrieving this “processed specification” as a callback during the compilation of the Gosling visualization. Through traversal of the Gosling specification, AltGosling separates the single tracks, the individual overlaid tracks, and overlaid multiple tracks to generate human-readable descriptions.

#### 2.4.2 Determining composition

From a visual standpoint, there is no difference regarding the composition or structure of the visualization between a singular track, two tracks that are overlaid, and a singular view with a singular track. We therefore use the separate term “chart” to identify the resulting individually drawn figures (see Supplementary Fig. S1 in the Supplementary Materials). Using the different alignments, arrangements, and layout properties of the individual tracks and views defined in the Gosling specification, we determine how the resulting charts are shown.

#### 2.4.3 Extracting chart types

A key element of the Grammar of Graphics (Wilkinson 2012) is that charts are created not by defining their type, such as a bar chart, but rather by combining low level visual elements to create the chart type. For example, a bar chart can be drawn using rectangular marks on a binned x-axis and a y-axis with the average quantitative value per binned area. However, alternative text best-practice guidelines from W3C include the chart type, and many BLV users find the chart type at the start of alternative text helpful (Jung *et al.* 2022). Therefore, we created a set of rules for determining known chart types from combinations of marks and visual elements (Table 1). The known chart table is a nonexhaustive list that we created based on visualization experience, drawing on Nusrat *et al.’*s taxonomy for genomics and Gosling’s example gallery (Nusrat *et al.* 2019, L’Yi *et al.* 2022). Then, for each chart in an AltGosling specification, we detect a chart type by comparing elements to these rules. If no known chart type is detected, we note the chart type as “chart with mark,” such as “chart with points.”

**Table 1.** Rules defined in AltGosling’s logic-based model for inferring chart types.^a^

Chart	Rules
“Scatter plot”	A track using a *point* mark, genomic *x*-axis, quantitative *y*-axis
“Line chart”	A track using a *line* mark, genomic *x*-axis, quantitative *y*-axis, or A track using a *line* mark, quantitative *x*-axis, genomic *y*-axis
“Bar chart”	A track using a *bar* mark, genomic *x*-axis, quantitative *y*-axis
“Heatmap”	A track using a *rect* mark, genomic *x*- and *xe*-axes, quantitative *color*
“Chromosome Ideogram”	A track using a *rect* mark, genomic *x*- and *xe*-axes, nominal *color*
“Matrix”	A track using a *bar* mark, genomic *x*- and *xe*-axes, genomic *y*- and *ye*-axes
“Chart with both horizontal and vertical lines”	A track using a *rule* mark, any types of *x*- and *y*-axes
“Chart with vertical lines”	A track using a *rule* mark, any type of *x*-axis without *y*-axis
“Chart with horizontal lines”	A track with a *rule* mark, any type of *y*-axis without *x*-axis
“Chart with [mark type]”	A track with a mark that does not have a detected chart type
“Circular [chart type]”	A track with any chart type that uses a circular layout
“Annotated [chart type]”	A track with multiple sub-tracks overlaid with at least one identified chart type

a This table describes the chart types that can be identified in AltGosling by looking up the use of visual encodings and other related properties in a Gosling specification. The *xe*-axis indicates the end position of a visual mark, often combined with the x-axis to create intervals.

Aside from chart type, BLV users highlighted the importance of axes (Jung *et al.* 2022). As people accessing the visualization might not be familiar with the meaning of the names of visual channels in Gosling (e.g. x1e representing the second end position on the *x*-axis) and marks (rect representing a rectangular mark), we created a mapping to natural language descriptions. In conveying the axes, we prioritized the *x*- and *y*-axes. For overlaid tracks with the same data source, we track which visual channels are combined with which marks. We keep the visual elements separated for overlaid tracks with different data sources.

Some blind users want to know the colors of a visualization to create a mental image, while others do not (Jung *et al.* 2022). Based on our co-design process, our final iteration does include information about what colors are used, importing default colors from the available themes. As the information is grouped under the “color” section, users that are not interested in this information can decide to skip this section.

#### 2.4.4 Retrieving data statistics

Information about the underlying data is a key element of a descriptive text (Jung *et al.* 2022). As part of the track traversal, AltGosling saves the data columns bound to different types of visual channels. Using the data columns, the data can be interpreted. We save the range of the genomic and quantitative channels and the genomic positions of the quantitative extrema. If present, we also save the genomic and quantitative range specific for each category in the nominal channels, including the category with the most extreme values. The retrieval of data statistics, like all properties, is kept flexible to extend information in future iterations of our tool.

### 2.5 Tree-structured navigable descriptions

The prevalent way of delivering alternative text is plain text. However, alternative text for complex images like genomics visualizations can become very long (a full description for a gene annotation is 73 sentences, as described in Section 3.1.2). Such long texts can be hard to navigate and have a high mental load. A tree structure is more easily navigable (Zong *et al.* 2022). Therefore, AltGosling creates a short description for the alt tag, a longer flat text description referenced in the longdesc-tag, and a hierarchical view of elements. The alt tag follows the principle of “chart type,” “type of data,” and “reason for including chart” based on W3C guidelines (summarized by https://medium.com/nightingale/writing-alt-text-for-data-visualization-2a218ef43f81). For a visualization with many charts, it only includes the list of chart types. The longer flat text description is a combination of chart-level and top-level information. For more than two charts, the description only includes an enumeration of chart types and titles, and users should refer to the navigable tree for more information.

**Figure 3.**
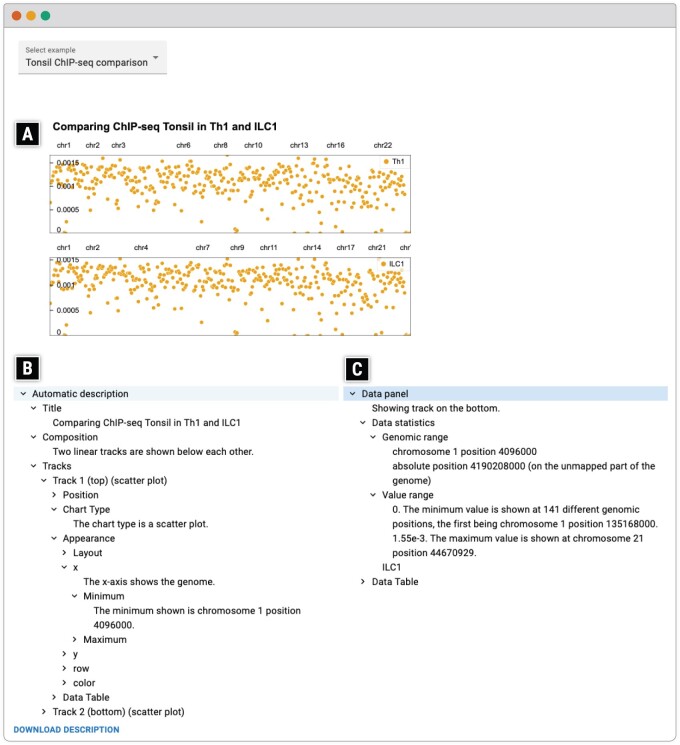
A public demo website of AltGosling. In addition to the (A) Gosling visualization, AltGosling presents two navigable hierarchical panels, (B) one containing information about the visualization (e.g. visual appearance and statistics), and (C) the other containing information about the most recently retrieved data (e.g. genomic range and corresponding data table). Users can select a different example using the drop-down menu on the left-top corner.

Novel in the area of alternative text, we propose a keyboard-navigable and screen reader accessible collapsible tree following HTML ARIA standard, similar to Olli, where the data is structured hierarchically (Fig. 3). For a visualization with multiple charts, the information is organized by chart.

The data is captured with each interaction and values are updated in the navigable tree. The state of collapsing is stored and remains. However, as navigation between different data sections of various charts becomes tedious, we also present a “data panel,” reducing the number of keyboard strokes to access the renewed data. This data panel includes the data statistics, and the full data table, as desired by many BLV users (Jung *et al.* 2022). With each data panel, we also determine the difference with the previously shown data panel and include a description of the difference.

Figure 3 shows how AltGosling shows the information panels. Using a screen reader, focusing on the first tree element, after expanding the first node, the options are “title,” “composition,” and “tracks.” After hearing the title and the composition, indicating that two charts are positioned above each other, a user might be specifically interested in the visual elements of the second chart. To retrieve these, the user needs to navigate to tracks, which contain “track top” and “track bottom,” the user can navigate into “track bottom” to then navigate e.g. to the *x*-axis of the bottom track.

This hierarchy with descriptions and detail nodes allows the user to ingest only the information they find important. This approach gives a high-level summary with the ability to drill down for details. If instead, all elements were directly listed, the user would need to go through all options to find the visual elements. In addition, for certain screen readers, a user cannot stop reading the text without escaping out of it. Therefore, breaking the information down in separate sections improves the user experience.

## 3 Results

### 3.1 Tree-structured navigable descriptions

**Figure 4.**
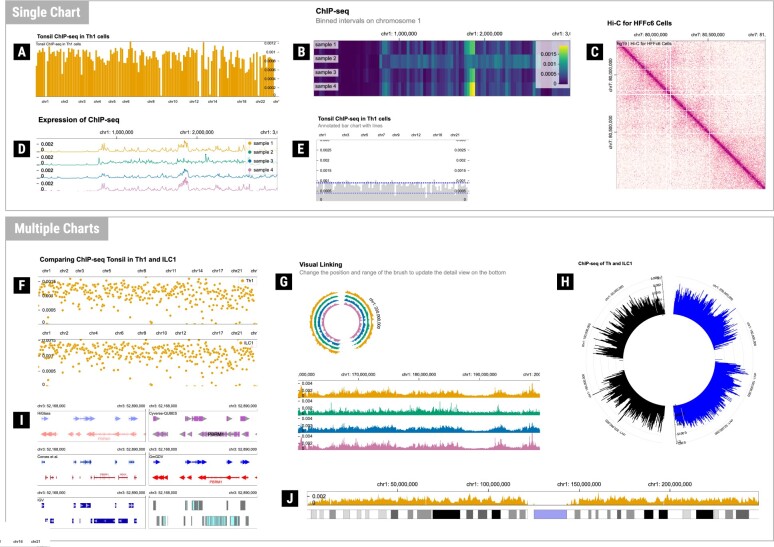
Examples of genomics data visualization supported with AltGosling. AltGosling can construct textual descriptions for a wide variety of genomics data visualizations, including single charts (A–E) and multiple chart compositions (F–J).

In this section, we use ten genomics data visualization examples (Fig. 4) to showcase the functionality of AltGosling in generating textual descriptions. Corresponding Gosling specifications and AltGosling descriptions are included in the Supplementary Materials. A video of a user navigating through the example in Fig. 4A with a screen reader is also included in the Supplementary Materials.

#### 3.1.1 Datasets

We constructed ten visualizations to showcase the functionality of AltGosling. The visualizations are chosen to include a range of functionalities of Gosling and be commonly used visualizations. The data for Fig. 4A–B, D–H, and J are an aggregate of ChIP-seq samples (GEO accession codes GSM2048305, GSM1375210, GSM2048292, GSM2048310) (Hammoud *et al.* 2014, Koues *et al.* 2016). Figure 4C uses Micro-C data (Krietenstein *et al.* 2020). Figure 4I uses data from the NCBI GenBank (GCA_000001405.15). Figure 4J additionally uses hg38 genome assembly from the UCSC Genome Browser (Kent *et al.* 2002). Figure 4A, F, and J were used in the co-design process.

#### 3.1.2 Alt text, long description, and tree-structured descriptions

AltGosling creates three variations of text descriptions. (i) The alt text, which is embedded with the alt-tag. (ii) The long description, which is referred to with the longdesc-tag. (iii) The tree-structured navigable descriptions, embedded as elements on a webpage. This section will focus on the panel containing all information, as the second data panel only focuses on the most recently interacted data track.

For the purpose of comparison, we also concatenated all information encaptured in the tree description as a flat text, which we refer to as the full description. In the case of one or two singular tracks, this “full description” is the same as the “long description.” With overlaid tracks or more than two tracks, the long description refers to the individual chart descriptions and is therefore shorter than the full description.

The lengths of the created alt texts vary between 6 and 19 words (mean = 11.2) and are always one sentence. The lengths of the created long descriptions vary between 9 and 244 words (mean = 130.8) with an average of 11.9 sentences. Notably, the shortest long description is that of an overlaid chart, which refers to the individual charts.

The lengths of the full descriptions vary between 35 and 848 words (mean = 222.4) with an average of 20.8 sentences. The longest full description (with 73 sentences) is for the gene annotation example, which has six charts. All full descriptions with multiple charts are longer than those with a single chart. The average lengths of the full descriptions for multiple charts is 332.4 words, while the average for single charts is 112.4 words. Given that a majority of screen reader users prefer the length of descriptive texts to be between 2 and 8 sentences (Jung *et al.* 2022), one can easily see the reason for a tree-navigable presentation.

#### 3.1.3 Text descriptions

We distinguish between views and tracks as designed in the Gosling specification, and charts, which are the individual visual canvases drawn in the Gosling visualization and seen by a user. AltGosling conveys information about the visual composition of Gosling, and therefore conveys these charts.

In Gosling, multiple tracks can be overlaid, i.e. drawn on top of each other. In AltGosling, overlaid tracks constitute one chart, as it’s a singular visual canvas (see Supplementary Fig. S1 in the Supplementary Materials for an additional explanation of this concept). Examples are shown in Fig. 4D, E, and I. With Gosling properties arrangement and alignment, as well as layout, AltGosling determines the composition of charts. Charts are referred to by their positioning. For example, the composition shown in Fig. 4I is described as follows: “There are 6 tracks. There are 3 rows. Each row has 2 tracks next to each other.” Then, the tracks are named “Track 1 (top row, left),” “Track 2 (second row, left),” etc. Whether a Gosling view makes up one or two charts is subjective in certain cases (see Supplementary Fig. S1 in the Supplementary Materials for an illustration). Adding multiple tracks which all share the same axes can be seen as a single chart or as multiple charts. AltGosling combines overlaid tracks into a single chart, but otherwise views separate tracks as separate charts, even if the visual appearance is closer to an individual chart, such as in Fig. 4H, where the composition is described as follows: “Two circular tracks form one ring together, each forming half of the ring.”

One key feature of AltGosling is that it can infer chart types from Gosling specifications. Figure 4B and J show this inference with a heatmap and ideogram. In Fig. 4E, the two overlaid tracks are described as a bar chart and a chart with horizontal lines. Together, they are described as an annotated bar chart. When chart type inference is impossible, such as the gene annotation track in the top left corner of Fig. 4I, the chart is described with the mark and if applicable, circularity, e.g. “chart with triangles.” With overlaid tracks, for each combination of mark, channels and layout, a chart type is inferred. These chart types are listed in order of the track determination. The left chart of the second row in Fig. 4I shows that this ordering is not necessarily informative: “Chart with text, chart with text, chart with rectangles, chart with rectangles, ideogram and chart with vertical lines.” In addition, with text annotation, AltGosling only detects that there is text, but not the content of the text.

Each description first mentions the *x* and *y*-axes, followed by other visual channels. Rather than directly mentioning the Gosling channel names (e.g. the “xe”-channel to indicate a second (often the end of a) genomic position, more descriptive words, such as “genomic intervals,” are used).

The genomic positions in the data are absolute positions on the reference genome. Our tool converts these to relative positions (including chromosome numbers). For example, position 1191936000 with reference genome hg38 is converted into chromosome 6, position 130737676. Due to variability in the used reference genomes, it is possible that certain genomic positions are unmapped, and therefore are not associated with a chromosome. As unmapped absolute positions are added at the end in Gosling, if AltGosling would simply state the minimum and maximum as ranges, the range would be from e.g. “chromosome 1 to an unknown chromosome.” Since this is unintuitive, our tool determines the range of chromosomes that is shown, such as in Fig. 4A: “The genomic range is shown from chromosome 1 to chromosome 22 and the X and Y chromosomes, as well as an unmapped part of the genome at the end”.

### 3.2 Feedback from the co-design process

As part of our evaluation, one fully blind co-author reviewed three distinct AltGosling examples (Fig. 4A, F, and J) to assess usefulness, completeness and usability. The feedback from the co-author can be grouped into three categories: (i) navigation, (ii) structure, and (iii) language. (i) They found AltGosling descriptions generally easy to navigate. The navigation on the tree-structured descriptions was fairly straightforward, and the full data table was accessible in the screen reader. Improvements were made to the pre-expansion of panels and the separation of navigation of panels. (ii) The separation of tracks in the panel was regarded as positive. Improvements were made to the organization of information in nodes to read specific data more easily. The concatenations of details included in earlier iterations were removed as this proved long segments with duplication of information. The grouping of information was adjusted to be more intuitive. (iii) Specific language was changed to be more comprehensible, specifically regarding colors. It was regarded as positive that charts were being referred to by position. The chart type was added to this referral. A detailed description of changes between the two iterations and reviews of the first iteration is included in the Supplementary Materials.

### 3.3 Comparison to artificial intelligence

We compared our tool to three image-based AI methods, two of which are image classifiers, and one is specifically designed to create alt text (Leotta *et al.* 2022). We uploaded Fig. 4A and F to the Amazon Rek Label Detection demo (v4, bucket rekognition-console-v4-prod-iad, us-east-1) (https://aws.amazon.com/rekognition/) and to the Google Cloud Vision AI demo (v1) (https://cloud.google.com/vision/). We also uploaded the same screenshots to AltText.AI (v1) (https://alttext.ai). All screenshots are included in the Supplementary Materials (Supplementary Figs S3–S8). Amazon Rek was able to establish that it's a chart (with >90% accuracy) and the type of chart (with >55% accuracy) within the first three labels. Google Cloud Vision AI could not determine the chart type and had ranging labels including Font, Rectangle, City, Organism, and Art. Of all three vision-based tools, AltText.AI was the most effective at creating alt text, able to identify the title, axes, and chart type.

We also used GPT-4o (version gpt-4o-2024-05-13) (OpenAI 2023), OpenAI’s current flagship model, to create alt text and longer text descriptions based on the Gosling specifications and images of Fig. 4A and F. The prompts and responses are included in Supplementary Materials. The created alt texts are very similar to those of AltGosling (GPT-4o: Tonsil ChIP-seq in Th1 cells bar chart; AltGosling: Bar chart showing genomic data, titled Tonsil ChIP-seq in Th1 cells). For the long descriptions, GPT-4o was able to detect visual encodings (Level 1 in the semantic model of Lundgard and Satyanarayan 2022), such as encodings, chart type, and axis ranges (though the axis ranges were based on the image labels, so the range was indicated to be up to chromosome 22, which was the last label, even though the chart also showed the genome beyond this). It also indicated the legends shown. GPT-4o created well-structured sentences, such as when explaining what the marks encode, utilizing contextual information from the title. This could help in understanding what is being shown. However, it did not produce any statistical information (Level 2 in this semantic model), such as extrema. GPT-4o is limited to the specification and image and does not have direct access to the underlying data. When asked to compare the two charts and describe the data of the example in Fig. 4F, it reiterated information from the Gosling specification. It also included a category in the description that was filtered with a data transform and therefore not shown in the chart.

In the example of Fig. 4F, GPT-4o split out the information between the two charts, making the information easier to digest. However, GPT-4o is still only able to provide information in a flat text format. In addition, GPT-4o also cuts off after a certain word limit, is variable in its output, and is not able to provide direct updates on interactions with the visualization.

## 4 Discussion

The goal of this work was to address accessibility issues for BLV users posed by visualizations of genomic data in web applications, data portals, and other resources that contain genomic data. We proposed to address this problem by building an automated solution that uses specifications for grammar-based genomics data visualizations constructed with Gosling as input and translates them into alt text and other descriptions of the visualizations and data shown. We focused on flexibility by mapping the Gosling specification to the AltGosling specification first. From this, we rendered both plain text descriptions and in-depth hierarchical views of the visualization and its data.

In its current form, our tool focuses on extracting information on visual encoding (Level 1) and statistical information (Level 2) properties (Lundgard and Satyanarayan 2022). It detects chart types and visual elements and groups this information by charts detected from individual and overlaid Gosling tracks and views. The evaluation of the approach by a blind screen reader user found that the navigable tree is a much better option than a long flat-text description. The screen reader user was generally able to deduce the overall appearance of the charts and retrieve values such as extrema, and a few changes have been made to make interaction and information retrieval better.

A clear limitation of our tool is the lack of informative data trends and comparisons corresponding to perceptual and cognitive information (Level 3) data, which is generally considered desirable by BLV users (Lundgard and Satyanarayan 2022). While our tool denotes extreme values and locations of such values, there is no overall description of data trends in individual charts, as well as a comparison between data trends of different charts. Automatically conveying data trends is complicated as the important trends depend on the intent of the visualization. Furthermore, not all data transformations are captured and conveyed, as some focus on data manipulation, whereas others focus on the manipulation of visual elements. Future iterations could support more specific cases of Gosling’s logic. One limitation beyond the control of AltGosling is how creators of visualizations use labels, as AltGosling relies on these. If labels are not informative, AltGosling’s descriptions will also be less effective.

Regardless, our tool is still an improvement on the current state, where there is no alternative text, and images cannot be detected or are identified as “blank,” “frame,” “graphic,” or “object” (Sharif *et al.* 2021). It is also more informative than image-based AI tools and LLMs. Access to the underlying data proves a big advantage to GPT-4o. As a deterministic algorithm, AltGosling always provides the same information for the same visualization. Our tool excels in feature extraction and data processing, but sentence flows are a potential weakness as a deterministic rule-based tool. LLMs excel in rewriting, and future research could focus on incorporating LLMs in our tool to reduce redundancy and create better text flows. This would, however, introduce a potential delay of descriptions, which is particularly problematic when the visualizations are interactive. Given the rapid evolution of the field of LLMs, it is important to continue testing as models improve.

As the feature extraction and web-page rendering logic in AltGosling are separate components, the presentation of the features is not limited to a hierarchical tree; a graph-based model could also be implemented (Elavsky *et al.* 2024). While the focus of this work is support for screen readers, AltGosling is also compatible with Braille devices (tested with Selvas BLV BrailleSense 6) as it is compliant with WCAG 2.1 standards.

In addition to increasing accessibility, including textual descriptions in visualizations makes them easier to search for in search engines, can help sighted users understand visualizations better, and this tool could even be expanded to easily generate figure captions.

As AltGosling relies on Gosling, Gosling’s accessibility affects AltGosling. Gosling lacks certain accessibility features, most notably the potential for keyboard interaction. Support for keyboard interactions in Gosling is an ongoing effort. Future efforts could additionally focus on adding the ability to increase contrast and text size as a user. These changes also improve the accessibility and usability of AltGosling.

Despite its limitations, we have begun to lay the groundwork for accessibility in the area of visual impairments for genomics and biomedical sciences with our context-aware accessibility tool. However, much work remains in the field of accessibility in the biomedical sciences. We want to draw attention to this issue, as the vast majority of biomedical resources fail accessibility standards, preventing people from entering the field.

## Supplementary Material

btae670_Supplementary_Data

## Data Availability

No new data was generated in this research. All Gosling and AltGosling descriptions are included in the Supplementary Materials.
