# Supplementary material for: AltGosling: automatic generation of text descriptions for accessible genomics data visualization
Source: Bioinformatics. 2024 Nov 14;40(12):btae670. doi: 10.1093/bioinformatics/btae670 (PMC11630506; doi:10.1093/bioinformatics/btae670)
Supplement: btae670_Supplementary_Data [file btae670_supplementary_data.zip › AltGoslingSupplementaryMaterials2.docx]

**Supplementary Materials 2**

# 1 Gosling and AltGosling Layout


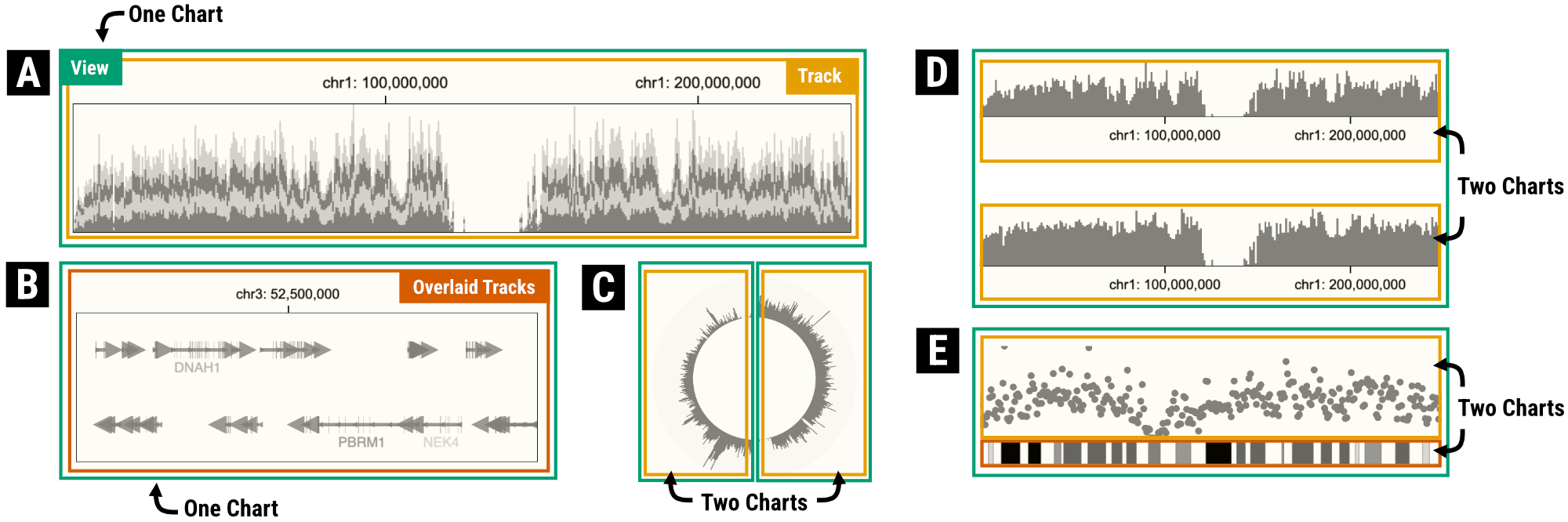


**Figure S1.** Illustration of mapping of view and chart properties in Gosling to charts in AltGosling. Gosling has views that can contain multiple tracks. A single track in a single view is one chart (A). Multiple overlaid tracks are also one chart (B). Multiple tracks that are not overlaid are two charts (C-E). In certain cases, from only the composition of views and tracks, it is unclear whether the visual canvas is closer to one or multiple charts. This is the case in circular visualizations (C). The space between tracks and the type of tracks can influence how it is perceived. When there is a large space between tracks and duplicate axes (D), it visually looks more like two separate charts than when this space is smaller and there is no additional axis (E). Both D and E are detected as two charts in AltGosling.

# 2 Co-design Reviews

The blind co-author reviewed the visualization examples in Figure 4A, F and J in-depth, and wrote these reviews in a document. Here, we provide a summary of their notes and subsequent discussions with other co-authors organized into three major themes: navigation, structure, and language.

## 2.1 Navigation

The navigation was generally easy and good. It was easy to arrow into various sections, everything was able to be read out and working. The data table was fully accessible in both reading and table modes of the screen reader. After updates, the opened sections will still be displayed and open and accessible. However, upon entering of the panels, part of this was already selected and expanded. This was confusing, as the co-author’s *“immediate assumption [was] that when a tree view is ‘expanded’, all the options are shown already, and you only need to arrow through them.”* Furthermore, the co-author noted that they needed to escape out of the data table to return to the rest of the information. *“After escaping the table mode in the data table, if you exit the page, there is a chance that all the sections will have closed.”* Additionally, the navigation of the two panels was not fully separated, where one can immediately jump from one panel to the next, which proved to be confusing. *“The ‘automatic description’ and ‘data panel’ options appear to ‘bleed’ into each other, rather than being self-contained. […] It’s a bit strange to realize that I’m viewing the ‘data panel’ options when I started from the ‘automatic description’.”*

## 2.2 Structure

Regarding structure, the organization of levels was easy to navigate. The similar organization with different examples made it easy to expect how the text was structured. The separation of tracks into their own sections was regarded as positive: *“I do like how the two tracks are each separated into their own sections; it makes differentiating between them very simple and clear.”* They were able to determine the appearance from its respective section, derive what the axes are showing, and find information such as extrema. The enumeration of genomic ranges in data statistics was structured well to be able to read individual data separately instead of reading it out all at once. *“It provided a nice feeling of separation and made reading and identifying specific data much easier in comparison to having everything read out all at once. It also saved on having to ‘escape’ out of the area in order to read any specifics of the text.”* For other information, this was not the case, and therefore read out in one instance. Additionally, due to the number of levels, navigating deeper into these became a bit cluttered. Adding the full descriptions at each node level was not regarded as positive. *“[The] description is really long, and while the screen-reader is able to read it all out in one go, it’s a lot to remember.”* The full descriptions also did not add any new information in respect to the detail sections. *“[The] biggest issue I found was a tendency towards repetition.”* This was exacerbated by larger examples, making for more repetition and longer descriptions. From this we conclude that the navigable tree creates a much better interaction than the long description. Another part of the feedback was aimed at the grouping of appearance and data description was not intuitive. The co-author indicated that it would have made more sense if the information on the minimum and maximum y-values was denoted under the appearance section that indicated that the y-axis was quantitative.

## 2.3 Language

Concerning the language used, the co-author indicated that it was positive that the sections clearly include the position of the referred track, and for the data panel *“it was [also] clear to which chart the data panel was referring”*. The channels were straightforward and used names that they were familiar with. The inclusion of the chart type was regarded as positive. They suggested to also include this in the distinction of tracks, e.g. that the top track is a bar chart. There was some information missing making it hard for the co-author to create a mental model, such as whether these visualizations were 2D or 3D, as well as the colors of the groups, which were not mentioned. Though the co-author cannot see color, since many charts they have previously interacted with included the actual colors, they found it easier to create a mental model if these were included. Regarding the phrasing of *“the color shows categories”*, the co-author suggested being more straightforward and using *“the bar chart is color coordinated”* or *“the bar color corresponds to the categories”*. Furthermore, long numbers, such as genomic positions, were hard to remember unless you went over them a few times. Regarding composition, the usage of ‘composition’ over ‘compositions’ was confusing. The difference between charts and tracks was also not clear, as well as the row channel, and how these are all distinct. *“It’s hard to know if the figure is made up of two charts, left and right, or two charts with one track each [...]. If it’s the first option, then I’m thinking that the ‘tracks’ are synonymous with the charts themselves, and there’s no difference between the terms. If it’s the second option, then the charts each have one track which corresponds to them.”*

## 2.4 Knowledge-specific Issues

A few issues that the co-author encountered are more specific to (a lack of) genomics knowledge. These included confusion about the difference between the genomic range and value range, what an ideogram was, and what the rectangles in an ideogram stand for: *“With the ‘rectangles’ representing categories, the closest chart I can think of it resembling is something like a flow or bar chart, with rectangular ‘boxes’ representing the categories in the flow chart, or the bars made to resemble rectangles or have rectangular ‘holes’ cut into them. How that would look, I have no idea.'* They also questioned how an area chart differs from a bar chart. As the range of user knowledge can change, this shows how we need to be precise in our wording.

## 2.5 Changes to the Algorithm

Based on the review of the outputs of the first iteration of AltGosling, we have made various changes to the algorithm (Figure S2). We changed how the two panels are grouped to avoid confusing interactions between them. Additionally, we set all nodes to be unselected and collapsed at the start. Regarding structure, we deleted the ‘description’ sections, and instead encaptured all information in the detail sections, reducing the amount of repetitions and levels (Figure S2, 1). We made all sections individually collapsible so a user can first determine the topic and read out each information on demand, rather than all at once (Figure S2, 3). We also restructured the information from a grammar of graphics perspective which separated encodings and data to a more intuitive grouping from a chart perspective (Figure S2, 5). Regarding language, we changed some of the descriptions that were identified as vague. We added information on 2D or 3D (Figure S2, 4), shortened numbers (Figure S2, 6), and included chart type in the chart sections (Figure S2, 2), as well as color information.

**
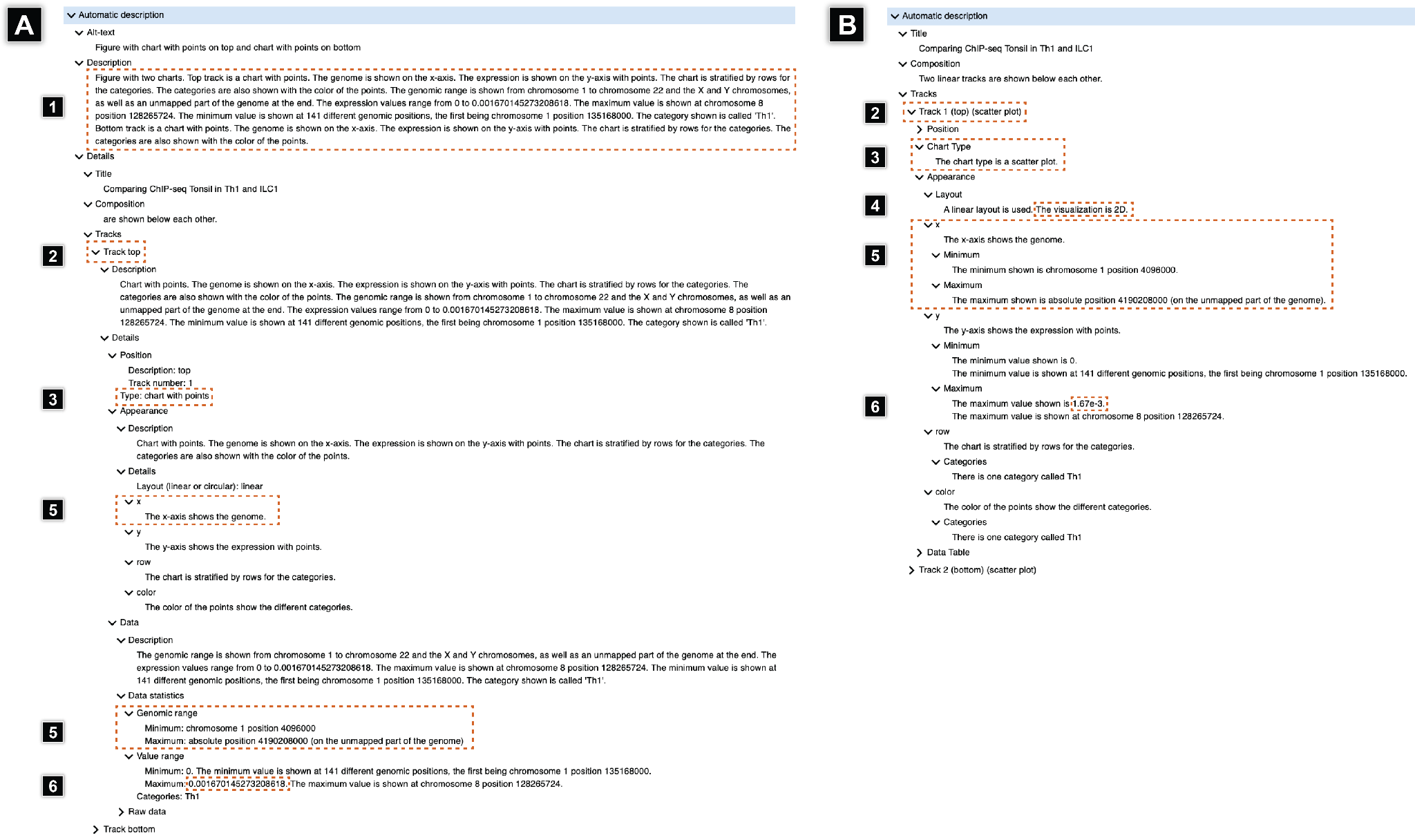
**

**Figure S2.** Changes to AltGosling based on co-design reviews, showing the output before (A) and after (B) these changes. Examples of a few changes are highlighted. Description sections were deleted (1), the track headers were changed to include chart types (2), all sections were made individually collapsible (3)., information on 2D/3D was added (4), information on encodings and their data was restructured (5), and values were shortened (6).

# 3 Comparison with AI Models

## 3.1 Amazon Rek


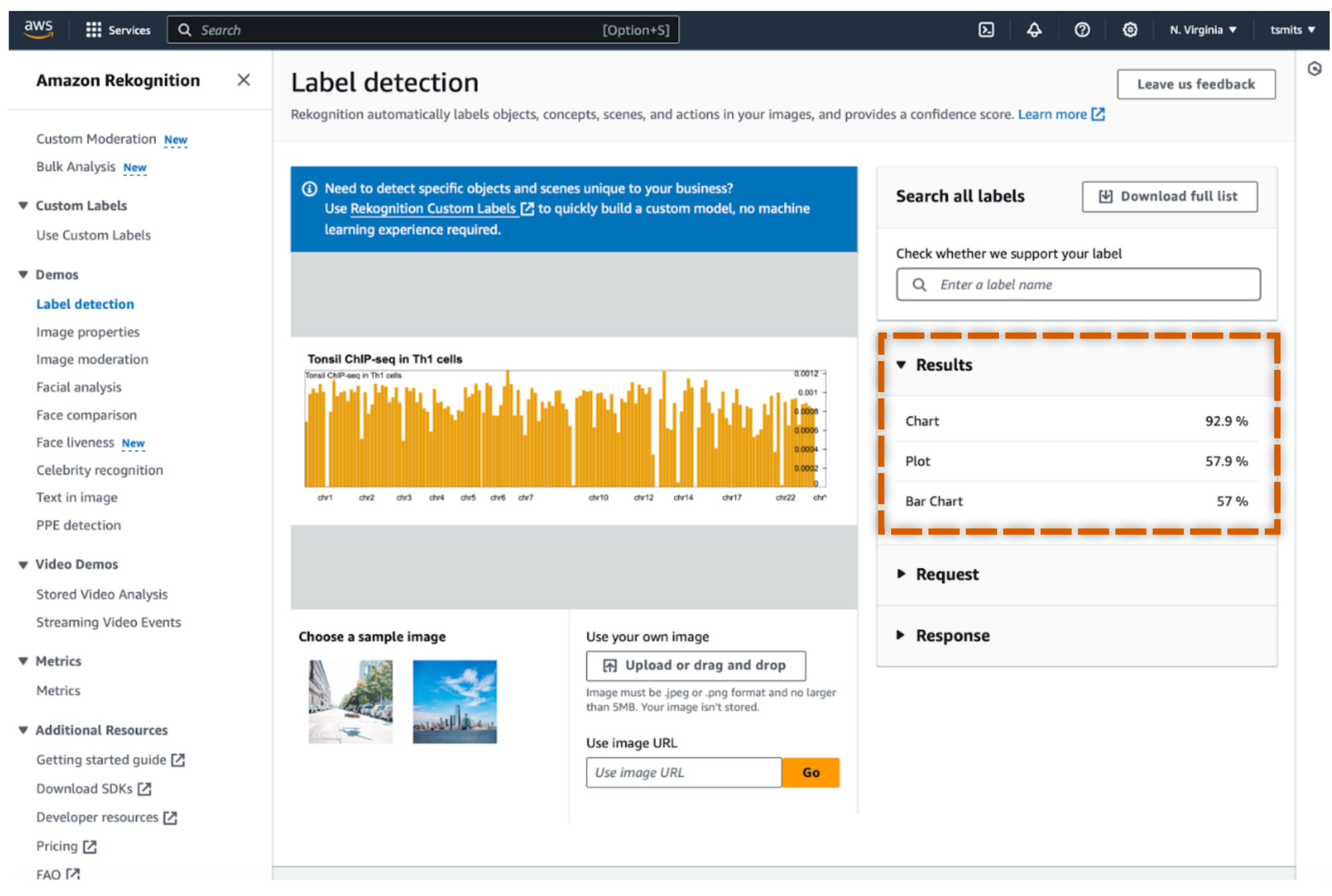


**Figure S3.** Screenshot of Amazon Rek Label Detection demo with a bar chart (Figure 4A). The detected labels are ‘Chart’ (92.9%), ‘Plot’ (57.9%) and ‘Bar Chart’ (57%).


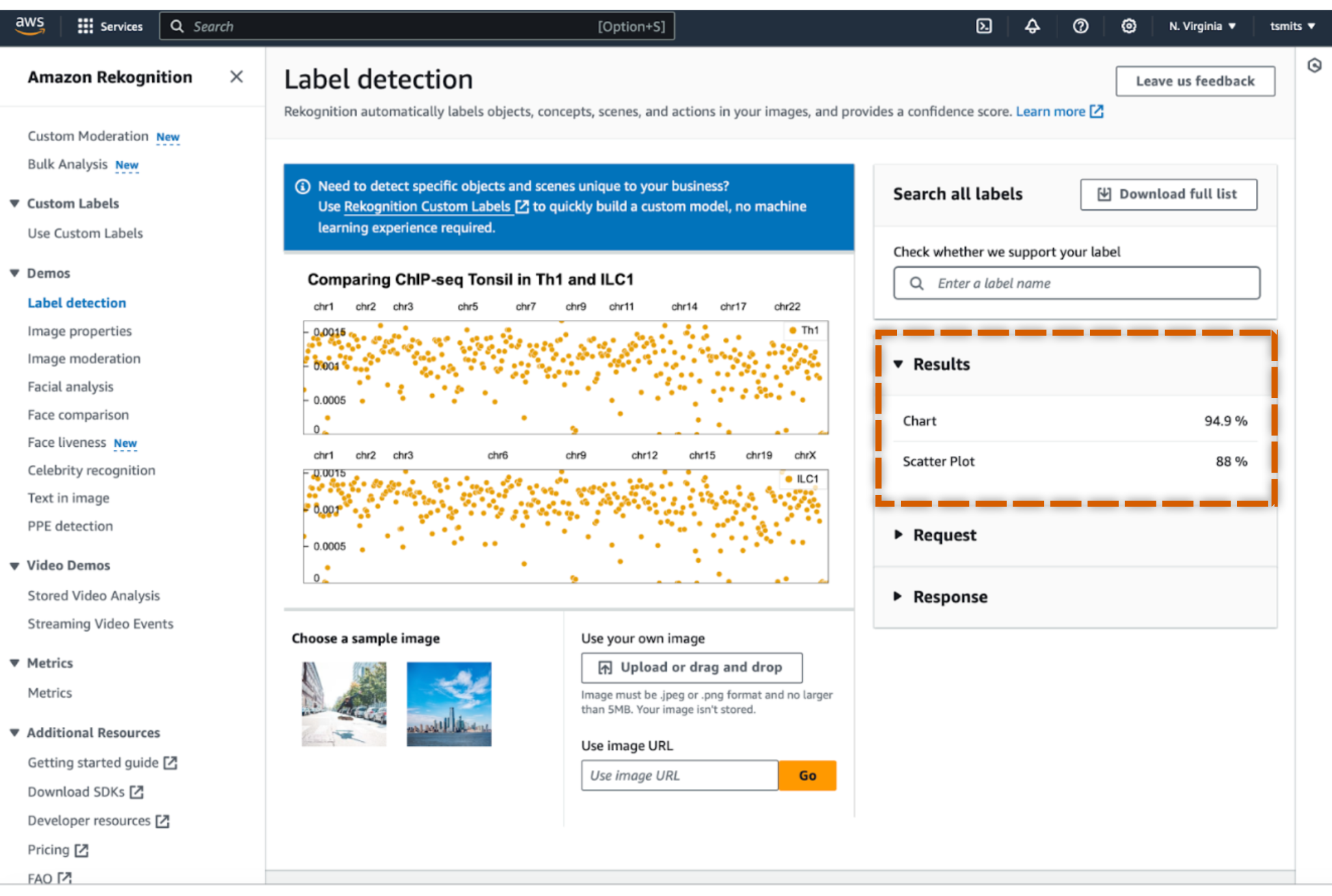


**Figure S4.** Screenshot of Amazon Rek Label Detection demo with two side-by-side scatter plots (Figure 4F). The detected labels are ‘Chart’ (94.9%) and ‘Scatter Plot’ (88%).

## 3.2 Google Cloud Vision AI


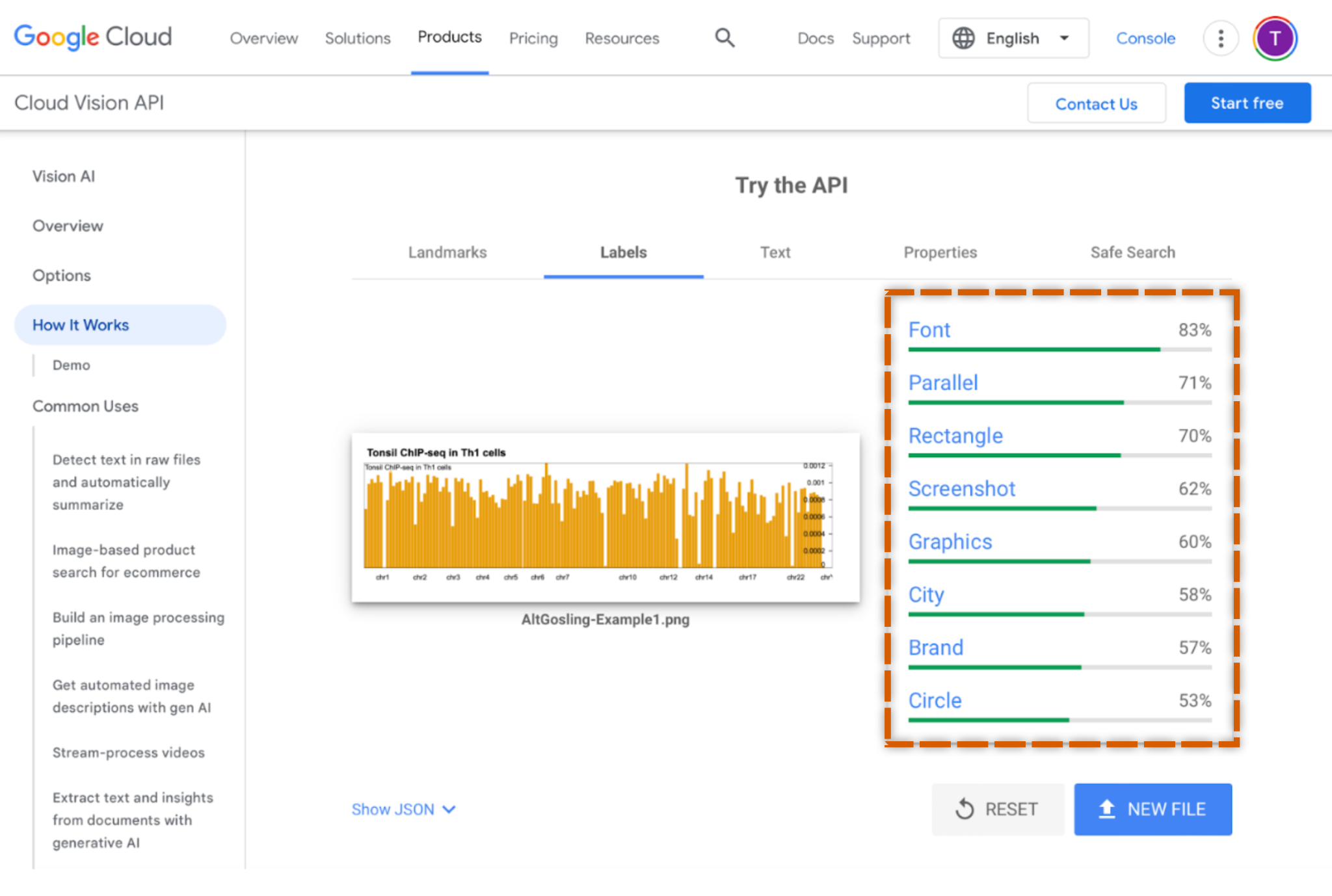
**Figure S5.** Screenshot of Google Cloud Vision AI demo with a bar chart (Figure 4A). The detected labels are ‘Font’ (83%), ‘Parallel’ (71%), ‘Rectangle’ (70%), ‘Screenshot’ (62%), ‘Graphics’ (60%), ‘City’ (58%), ‘Brand’ (57%) and ‘Circle’ (53%).


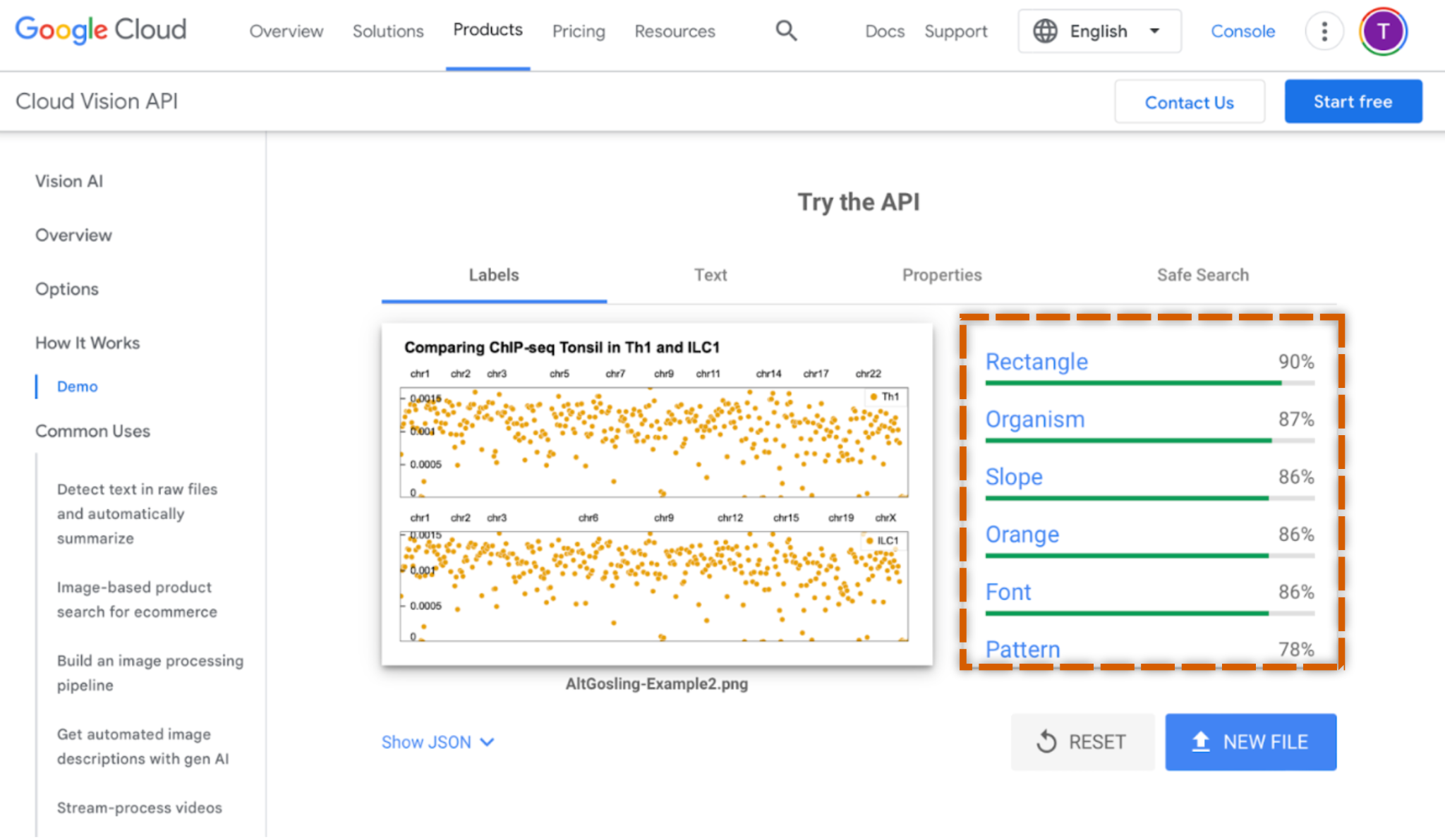
**Figure S6.** Screenshot of Google Cloud Vision AI demo with two side-by-side scatter plots (Figure 4F). The detected labels are ‘Rectangle’ (90%), Organism (87%), Slope (86%), Orange (86%), Font (86%), ‘Pattern’ (78%), ‘Parallel’ (78%), ‘Circle’ (74%), ‘Art’ (72%), ‘Illustration’ (69%), ‘Design’ (68%), ‘Screenshot’ (67%), ‘Symmetry’ (64%), ‘Graphics’ (63%), ‘Plot’ (62%), ‘Number’ (61%), ‘Diagram’ (57%), ‘Science’ (53%) and ‘Electric Blue’ (51%).

## 3.3 AltText.AI Alt Text Generator


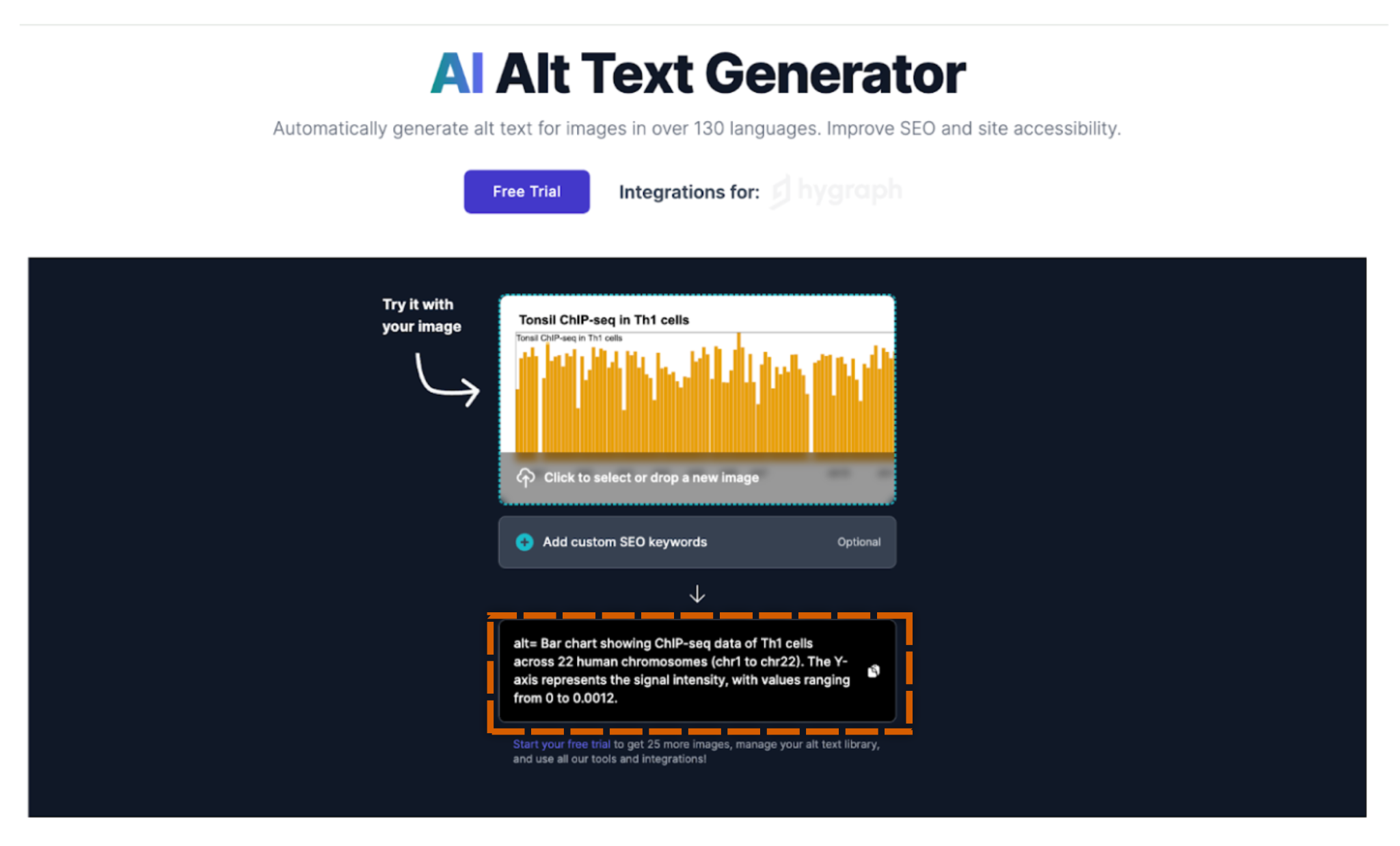


**Figure S7.** Screenshot of AIText.AI Alt Text Generator demo with a bar chart (Figure 4A). The generated alt text is ‘Bar chart showing ChIP-seq data of Th1 cells across 22 human chromosomes (chr1 to chr22). The Y-axis represents the signal intensity, with values ranging from 0 to 0.0012.’

**
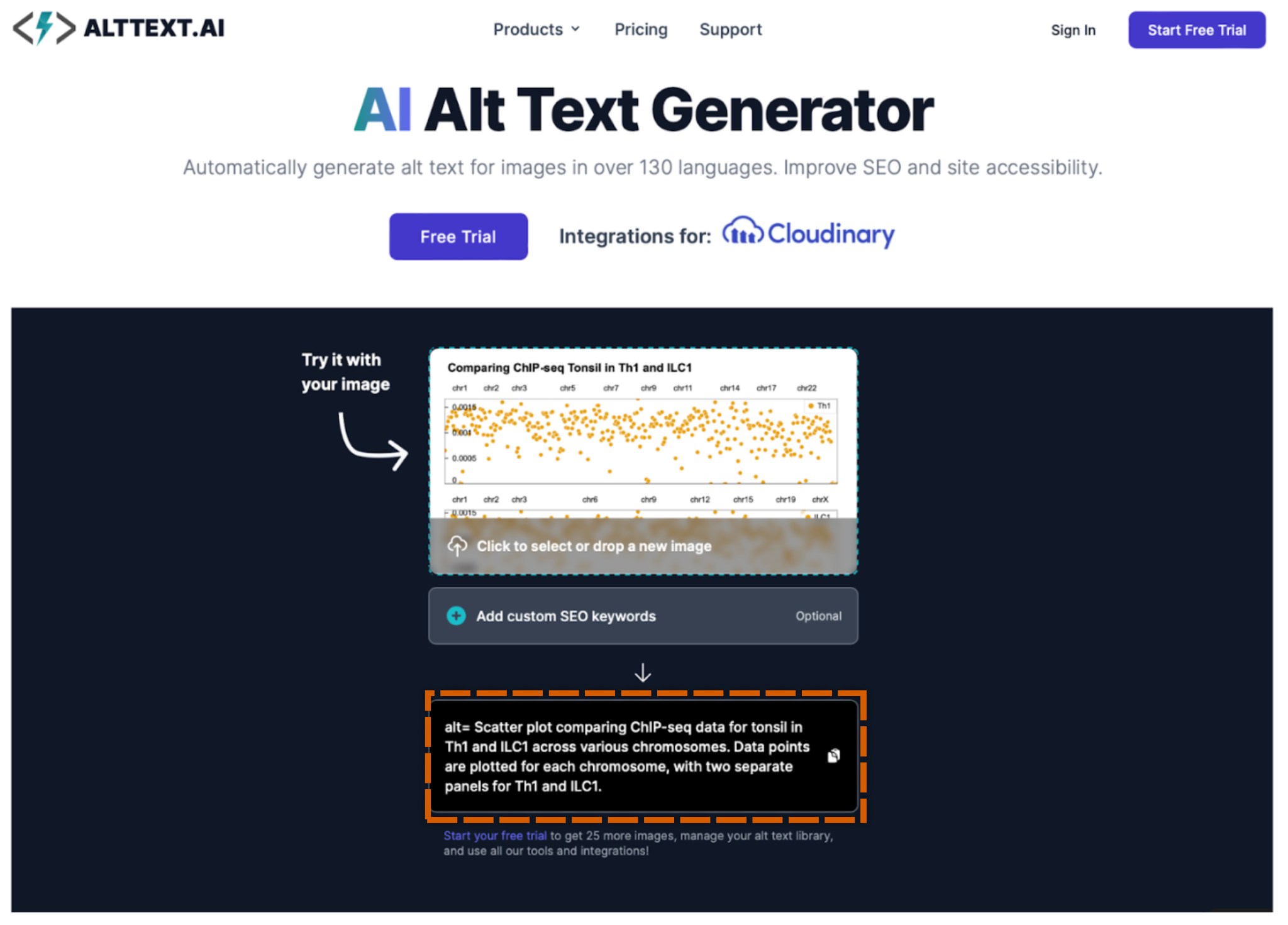
Figure S8.** Screenshot of AIText.AI Alt Text Generator demo with two side-by-side scatter plots (Figure 4F). The generated alt text is ‘Scatter plot comparing ChIP-seq data for tonsil in Th1 and ILC1 across various chromosomes. Data points are plotted for each chromosome, with two separate panels for Th1 and ILC1.’

##

## 3.4 GPT-4o

### 3.4.1 Example A (Bar Chart)

#### 3.4.1.1 Prompt

Given the following gosling specification and accompanying image, can you write me a short alt text and a long description for visually impaired people

{

"title": "Tonsil ChIP-seq in Th1 cells",

"tracks": [

{

"layout": "linear",

"width": 800,

"height": 180,

"data": {

"url": "https://resgen.io/api/v1/tileset_info/?d=UvVPeLHuRDiYA3qwFlm7xQ",

"type": "multivec",

"row": "sample",

"column": "position",

"value": "peak",

"categories": ["sample 1"],

"binSize": 5

},

"mark": "bar",

"x": {"field": "start", "type": "genomic", "axis": "bottom"},

"xe": {"field": "end", "type": "genomic"},

"y": {"field": "peak", "type": "quantitative", "axis": "right"},

"size": {"value": 5}

}

]

}

*Accompanying image:*


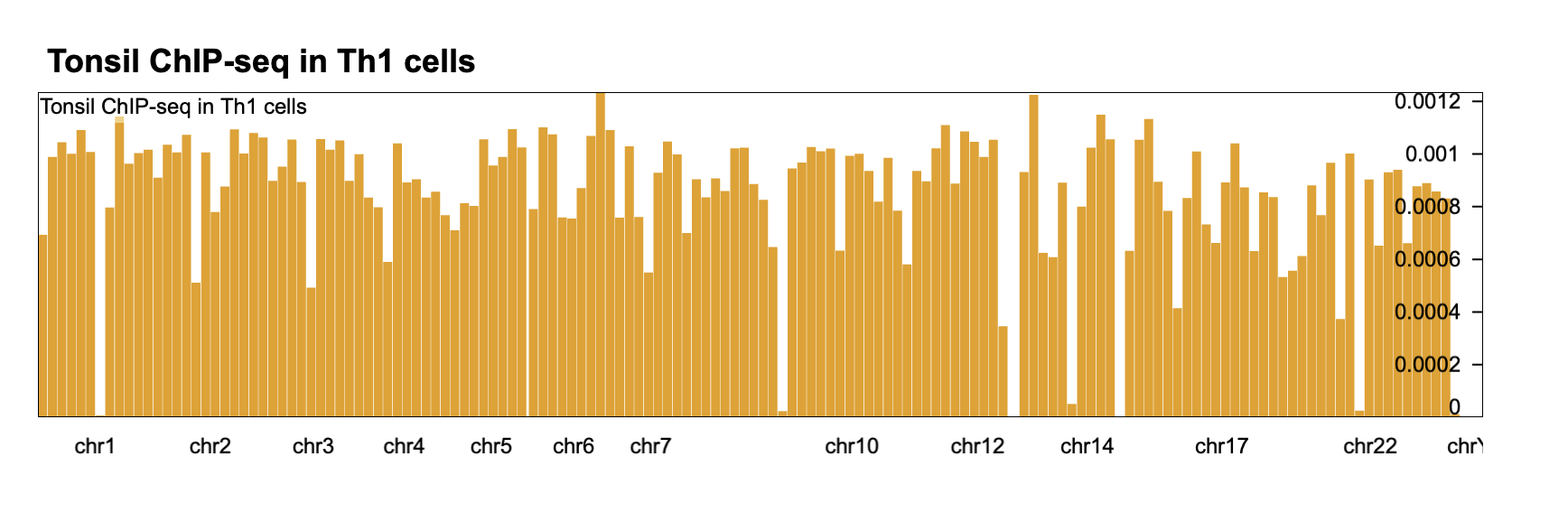


#### 3.4.1.2 Response

**Alt Text:**

"Tonsil ChIP-seq in Th1 cells bar chart."

**Long Description:**

This image shows a bar chart titled "Tonsil ChIP-seq in Th1 cells." The x-axis, labeled genomic 'position,' is marked with chromosome numbers from 'chr1' to 'chrY.' The y-axis, labeled 'peak,' measures quantitative values ranging from 0 to 0.0012. Each bar, representing the peak intensity value for a given genomic position, is colored in orange. The bar heights vary, showing different levels of peak intensity across the chromosomes. The chart provides a visual representation of ChIP-seq data specifically for tonsil Th1 cells, illustrating areas of chromatin immunoprecipitation across various genomic positions.

### 3.4.2 Example F (Comparison of Two Samples)

#### 3.4.2.1 Prompt

Prompts: Given the following gosling specification and accompanying image, can you write me a short alt text and a long description for visually impaired people

{

"title": "Comparing ChIP-seq Tonsil in Th1 and ILC1",

"layout": "linear",

"arrangement": "vertical",

"views": [

{

"tracks": [

{

"id": "track-1",

"data": {

"url": "https://server.gosling-lang.org/api/v1/tileset_info/?d=cistrome-multivec",

"type": "multivec",

"row": "sample",

"column": "position",

"value": "peak",

"categories": ["Th1"]

},

"mark": "point",

"x": {"field": "position", "type": "genomic", "axis": "top"},

"y": {"field": "peak", "type": "quantitative"},

"row": {"field": "sample", "type": "nominal"},

"color": {"field": "sample", "type": "nominal", "legend": true},

"stroke": {"value": "white"},

"strokeWidth": {"value": 0.5},

"tooltip": [

{"field": "start", "type": "genomic", "alt": "Start Position"},

{"field": "end", "type": "genomic", "alt": "End Position"},

{

"field": "peak",

"type": "quantitative",

"alt": "Value",

"format": ".2"

},

{"field": "sample", "type": "nominal", "alt": "Sample"}

],

"width": 600,

"height": 130

}

]

},

{

"tracks": [

{

"id": "track-2",

"data": {

"url": "https://server.gosling-lang.org/api/v1/tileset_info/?d=cistrome-multivec",

"type": "multivec",

"row": "sample",

"column": "position",

"value": "peak",

"categories": ["Th1", "Ununsed", "ILC1"]

},

"dataTransform": [{

"type": "filter", "field": "sample", "oneOf": ["ILC1"]

}],

"mark": "point",

"x": {"field": "position", "type": "genomic", "axis": "top"},

"y": {"field": "peak", "type": "quantitative"},

"row": {"field": "sample", "type": "nominal"},

"color": {"field": "sample", "type": "nominal", "legend": true},

"stroke": {"value": "white"},

"strokeWidth": {"value": 0.5},

"tooltip": [

{"field": "start", "type": "genomic", "alt": "Start Position"},

{"field": "end", "type": "genomic", "alt": "End Position"},

{

"field": "peak",

"type": "quantitative",

"alt": "Value",

"format": ".2"

},

{"field": "sample", "type": "nominal", "alt": "Sample"}

],

"width": 600,

"height": 130

}

]

}

]

}

*Accompanying image:*


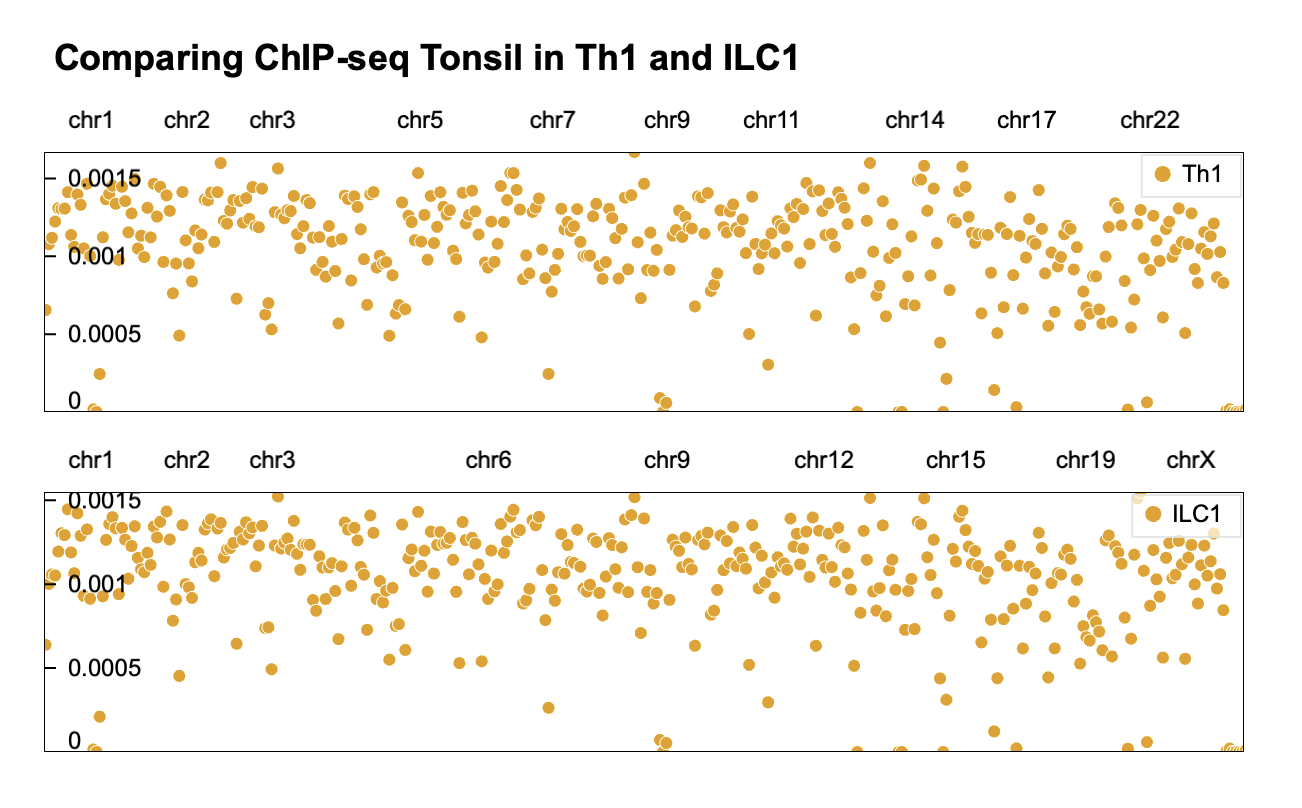


#### 3.4.2.2 Response

**Alt Text**

A scatter plot comparing ChIP-seq data for tonsil samples in Th1 and ILC1.

**Long Description**

The image displays two vertical scatter plots comparing ChIP-seq data for tonsil samples labeled Th1 and ILC1. The title at the top reads "Comparing ChIP-seq Tonsil in Th1 and ILC1."

1. **Top Scatter Plot (Th1):**

- X-Axis: Genomic positions labeled with chromosomal regions (chr1 to chr22).
- Y-Axis: Peak values ranging from 0 to 0.0015.
- Data Points: A dense scatter of small orange points indicating the presence of ChIP-seq peaks throughout different genomic regions for Th1 samples.
- Annotation: A legend in the top right corner indicates orange points represent Th1.

2. **Bottom Scatter Plot (ILC1):**

- X-Axis: Genomic positions labeled with chromosomal regions (chr1 to chrX).
- Y-Axis: Peak values ranging from 0 to 0.0015.
- Data Points: Another dense scatter of small orange points indicating ChIP-seq peaks for ILC1 samples in different genomic regions.
- Annotation: A legend in the bottom right corner shows orange points represent ILC1.

Both plots use the same scale for the X and Y-axes to facilitate comparison between the Th1 and ILC1 samples

#### 3.4.2.3 Prompt

Given the following gosling specification and accompanying image, can you describe the difference between the two charts

*Spec and image same as in 3.4.2.2, omitted for length.*

#### 3.4.2.4 Response

Response: The provided specification and the image both display point plots for ChIP-seq data from tonsil samples of Th1 and ILC1, arranged in a vertical layout. Here's a breakdown of the differences between the two charts:

1. **Data Source and Filtering**:

- **First Chart (Th1)**: Displays data for the Th1 sample.
  - **Data URL**: https://server.gosling-lang.org/api/v1/tileset_info/?d=cistrome-multivec
  - **Categories**: Filtered to include only Th1.
- **Second Chart (ILC1)**: Displays data for the ILC1 sample.
  - **Data URL**: https://server.gosling-lang.org/api/v1/tileset_info/?d=cistrome-multivec
  - **Categories**: Initially includes Th1, Ununsed, and ILC1, but then filtered to show only ILC1 through `dataTransform`.

2. **Axis and Data Representation**:

- Both charts have identical axes, with genomic positions along the x-axis and peak values along the y-axis.

3. **Color and Labeling**:

- **First Chart**: The color represents Th1, and the legend indicates "Th1".
- **Second Chart**: The color represents ILC1, and the legend indicates "ILC1".

4. **Tooltip Information**:

- Both charts

#### 3.4.2.5 Prompt

Given the following gosling specification and accompanying image, can you describe the data of the two charts

*Spec and image same as in 3.4.2.2, omitted for length.*

#### 3.4.2.6 Response

The provided Gosling specification defines a comparative visualization of ChIP-seq (Chromatin Immunoprecipitation Sequencing) data between Th1 (T helper 1 cells) and ILC1 (type 1 innate lymphoid cells) in tonsil samples, displayed in a vertical arrangement with two linear charts.

Here's a detailed description of the data depicted in the charts:

**General Layout**

- **Title**: "Comparing ChIP-seq Tonsil in Th1 and ILC1"

- **Arrangement**: Vertical (one chart on top of the other)

- **Width and Height of Each Track**: 600 x 130 units

**Upper Chart (Th1 Sample)**

- **Data Source**: https://server.gosling-lang.org/api/v1/tileset_info/?d=cistrome-multivec
- **Categories**: ["Th1"]
- **Data Fields**:
  - **Row**: "sample"
  - **Column**: "position" (genomic position on chromosomes)
  - **Value**: "peak" (height of the peaks in the ChIP-seq data)
  - **Position**: Genomic position along the x-axis
  - **Peak Value**: Quantitative (y-axis)
- **Visual Marks**: Points
  - **Color**: Based on "sample" (legend displayed with Th1 represented in orange)
  - **Stroke and Stroke Width**:
